# EFFECTS OF DEPRESSIVE SYMPTOMS ON COGNITIVE BEHAVIORAL INTERVENTIONS WITH CUSTODIAL GRANDMOTHERS

**DOI:** 10.1093/geroni/igac059.1690

**Published:** 2022-12-20

**Authors:** Carol Musil, Alexandra Jeanblanc, Christopher Burant, Jaclene Zausniewski

**Affiliations:** Case Western Reserve University, Cleveland, Ohio, United States; Case Western Reserve University, Cleveland, Ohio, United States; Case Western Reserve University, Parma, Ohio, United States; Case Western Reserve University, Cleveland, Ohio, United States

## Abstract

Over 25% of custodial grandmothers report elevated depressive symptoms, which may affect situational appraisals and learning/retaining new information, and thus the effectiveness of cognitive-behavioral interventions. We examined the effect of depressive symptoms on self-appraised stress and reward associated with caregiving to co-residential grandchildren in a sample of 342 grandmothers who participated in a nationwide behavioral RCT testing two methods of stress reduction. Participants completed a baseline questionnaire, one of 2 cognitive-behavioral interventions (guided journaling or journaling with Resourcefulness Skills Training ©), and 3 subsequent questionnaires over 6 months. We analyzed self-appraised stress and reward with RM-ANOVAs to evaluate whether level of depressive symptoms (CES-D below-16, 16-29, 30+) affected the effectiveness of each arm with regard to self-appraised stress and reward. We found differences by depressive symptom category and interaction effects of depressive symptoms by arm in self-appraised stress. The below-16 group had relatively constant appraised stress over time, but the 16-29 and 30+ groups had significant decreases in stress over time. The Resourcefulness Skills Training © group had an average decrease in stress of .68 over time compared to .2 points for the journal-only group; there were no intervention effects in the below-16 group. The highest symptom group showed a stronger effect for the journal-only intervention, perhaps reflecting the challenges in learning and applying new skills. Self-appraised reward increased over time across groups. Participants' level of depressive symptoms have significant effects on the effectiveness of interventions and should be taken into consideration when designing studies and analyzing effectiveness.

